# An interactomics overview of the human and bovine milk proteome over lactation

**DOI:** 10.1186/s12953-016-0110-0

**Published:** 2017-01-05

**Authors:** Lina Zhang, Aalt D. J. van Dijk, Kasper Hettinga

**Affiliations:** 1 Dairy Science and Technology, Food Quality and Design Group, Wageningen University, Postbox 8129, 6700EV Wageningen, The Netherlands; 2 Biometris, Wageningen University and Research Centre, P.O. Box 16, 6700 AA Wageningen, The Netherlands; 3 Bioinformatics Group, Wageningen University, Droevendaalsesteeg 1, 6708 PB Wageningen, The Netherlands; 4 Bioscience, cluster Applied Bioinformatics, Wageningen University and Research, Droevendaalsesteeg 1, 6708 PB Wageningen, The Netherlands

**Keywords:** Proteomics, Protein interaction networks, Lactation, Human milk, Bovine milk

## Abstract

**Background:**

Milk is the most important food for growth and development of the neonate, because of its nutrient composition and presence of many bioactive proteins. Differences between human and bovine milk in low abundant proteins have not been extensively studied. To better understand the differences between human and bovine milk, the qualitative and quantitative differences in the milk proteome as well as their changes over lactation were compared using both label-free and labelled proteomics techniques. These datasets were analysed and compared, to better understand the role of milk proteins in development of the newborn.

**Methods:**

Human and bovine milk samples were prepared by using filter-aided sample preparation (FASP) combined with dimethyl labelling and analysed by nano LC LTQ-Orbitrap XL mass spectrometry.

**Results:**

The human and bovine milk proteome show similarities with regard to the distribution over biological functions, especially the dominant presence of enzymes, transport and immune-related proteins. At a quantitative level, the human and bovine milk proteome differed not only between species but also over lactation within species. Dominant enzymes that differed between species were those assisting in nutrient digestion, with bile salt-activated lipase being abundant in human milk and pancreatic ribonuclease being abundant in bovine milk. As lactation advances, immune-related proteins decreased slower in human milk compared to bovine milk. Notwithstanding these quantitative differences, analysis of human and bovine co-expression networks and protein-protein interaction networks indicated that a subset of milk proteins displayed highly similar interactions in each of the different networks, which may be related to the general importance of milk in nutrition and healthy development of the newborn.

**Conclusions:**

Our findings promote a better understanding of the differences and similarities in dynamics of human and bovine milk proteins, thereby also providing guidance for further improvement of infant formula.

**Electronic supplementary material:**

The online version of this article (doi:10.1186/s12953-016-0110-0) contains supplementary material, which is available to authorized users.

## Background

Milk is one of the richest foods, as it provides complete nutrition and bioactive components for healthy development of the newborn. These nutritional and bioactive components are essential for the neonate, for example for cognitive development, pathogen prevention, intestinal microflora modulation, and development of the immune system [1, 2]. Of these bioactive components, proteins have attracted great attention because of their importance in the protection of the neonate. With the development of proteomics techniques, more and more proteins, including both high and low abundant proteins, were characterized in the last few decades [3–5].

However, milk proteins are variable in presence and concentration due to many factors. One of the most obvious factors causing differences in protein concentration is species differences [6]. Caseins accounts for 80% (w/w) of the bovine milk proteins, and for 50% of human milk proteins [4]. In addition, β-lactoglobulin exists in bovine milk but cannot be found in human milk [6, 7]. Human and bovine milk diverge not only in their high abundant protein composition, but also in their low abundant protein composition. A total of 268 and 269 proteins were previously identified in human and bovine milk, respectively, in our previous study [8]. Of these proteins, 44 from human milk and 51 from bovine milk were related to the host defense system. Specifically, the concentration of proteins involved in the mucosal immune system, immunoglobulin A, CD14, lactoferrin, and lysozyme, were present in much higher concentration in human milk than bovine milk [8].

Furthermore, milk proteins also differ in concentration over lactation. Immunoglobulins have been reported to change rapidly in concentration from colostrum to mature milk in both human [9, 10] and bovine milk [11–13]. Moreover, the low abundant proteins, such as complement proteins, lipid synthesis and transport proteins, and enzymes were also reported to change as lactation advances [14, 15]. However, the differences in changes of proteins over lactation has not been reported between human milk and bovine milk directly, although we reported the changes in the species separately [13, 16–18].

As human milk is used as reference and bovine milk is used as protein source for producing infant formula [19], the differences in the health outcomes between breastfed and formula-fed infants could be related to the differences in the nutrient intake [6]. Breastfed infants were reported to have fewer infections (gastrointestinal infections, acute otitis media), reduced risk for celiac disease, obesity, and diabetes compared to formula-fed infants [19]. Therefore, the aim of this study is to better understand the role of different proteins, especially those involved in immune activity, in both human milk and bovine milk through elaborating the existing data in qualitative and quantitative proteome [8] and their changes over lactation [13, 16–18]. Separate interactomics studies of human and bovine milk proteins have previously been performed, using published data collected from many different sources [20, 21]. In this study, the analysis is a comparative data analysis on both species simultaneously, where data has been collected on a single instrument [8, 13, 16–18], throughout lactation, allowing a better comparison between species.

In the current study, the human and bovine milk data in Data set 1 [8] was reanalysed by Maxquant to give a more precise comparison in the quantitative differences between human and bovine milk proteins. The changes of both human and bovine milk proteome over lactation in Data set 2 [13, 16–18] were reanalysed using a co-expression (expression meaning the relative abundance) network approach and integrated with protein-protein interaction network data. The additional analysis enhances the comparison between human and bovine milk proteome from both qualitative and quantitative differences in milk proteome and their differences in changes over lactation. This should contribute to better understanding of the differences and similarities in biological functions networks of proteins, especially with regard to immune activity, in both the human and bovine milk proteome.

## Result

**Fig. 1:**
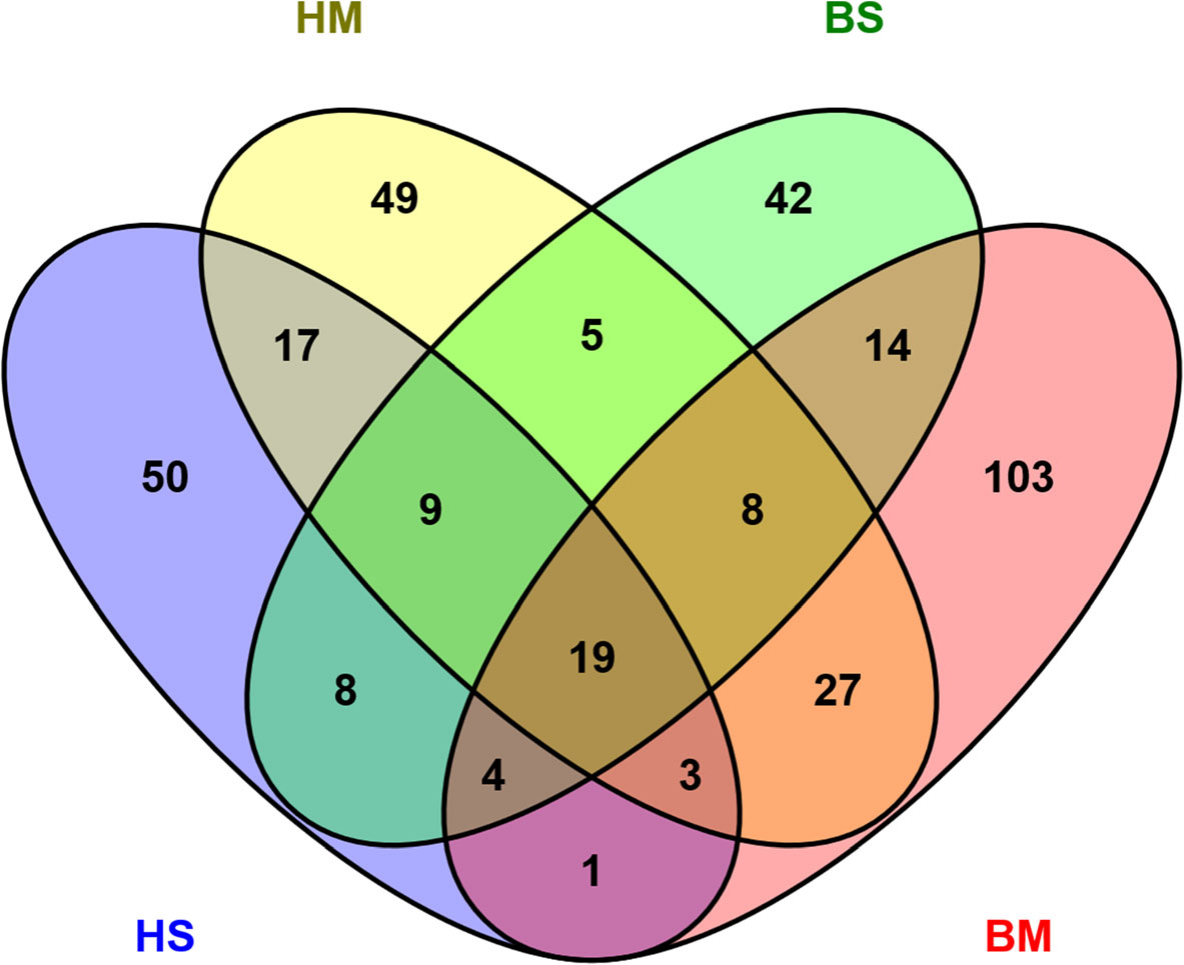
The number of identified proteins in human milk and bovine milk (HS is human serum protein; HM is human milk fat globule membrane (MFGM) protein; BS is bovine serum protein; BM is bovine MFGM; identified number of proteins in HS, HM, BS, BM are 111. 137, 109, 179)

**Fig. 2:**
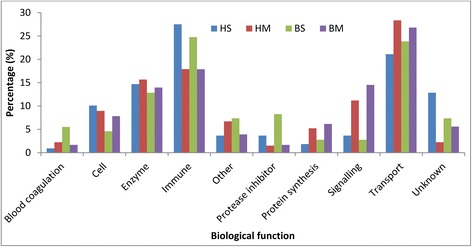
The distribution of biological functions found in human and bovine milk ((HS is human serum protein; HM is human MFGM protein; BS is bovine serum protein; BM is bovine MFGM)

A total of 379 proteins were quantified through reanalyzing the human and bovine milk of data set 1 prepared by filter-aided sample preparation (FASP) and LC-MS/MS. The specific number of identified proteins in milk fat globule membrane (MFGM) and milk serum proteins for both human and bovine species are shown in Fig. 1. Of these quantified proteins, 93 proteins present in both species. Figure 2 shows that both human milk and bovine milk have similar distribution over biological functions in quantified MFGM and milk serum proteins. Transport proteins, enzymes, and immune-related proteins were the three dominant biological function groups in both human and bovine milk (Fig. 2). The biological enrichment of these three protein groups were shown in Additional file 1: Table S1. However, the number of proteins in these three dominant groups was different between human and bovine milk. Bovine milk contained a higher number of transport proteins than human milk (Fig. 2), which was dominated by lipid and protein transporters. Although the number of enzymes were similar, they were quite different in the type between human and bovine milk. The enzymes assisting nutrient digestion were bile salt-activated lipase (CEL), and lipoprotein lipase (LPL) alpha-trypsin chain 1 (PRSS1) in human milk (Table 1) [16, 18], whereas pancreatic ribonuclease 1 (RNASE1), LPL, and ribonuclease 4 (RNASE4) were dominant in bovine milk [13, 17].

**Table 1:** Quantitative comparison of overlap human milk MFGM and bovine milk MFGM (Bold proteins are significantly different proteins by one-way ANOVA; the values are log10 average iBAQ values of proteins; Human milk samples were collected from 10 healthy mothers that were between 3 and 10 months in lactation; Bovine milk samples were collected from 30 clinically healthy cows that were between 3 weeks and 10 months in lactation; data was normally distributed)

Gene name	Protein name	HM	CM	Log_2_ (HM/CM)	*P* value
**LTF**	**Lactotransferrin**	6.87	5.03	6.13	0.000
**ALB**	**Serum albumin**	6.02	4.46	5.18	0.000
**FOLR1**	**Folate receptor alpha**	5.16	3.85	4.35	0.000
**CD14**	**Monocyte differentiation antigen CD14**	5.37	4.51	2.87	0.000
**LALBA**	**Alpha-lactalbumin**	7.16	6.44	2.38	0.001
**TLR2**	**Toll-like receptor 2**	4.30	3.70	2.01	0.008
KRT79	Keratin, type II cytoskeletal 79	4.47	4.10	1.23	0.586
ATP2B2	Plasma membrane calcium-transporting ATPase 2	3.38	3.10	0.95	0.164
YWHAZ	14-3-3 protein zeta/delta	4.29	4.02	0.89	0.084
FASN	Fatty acid synthase	4.02	3.81	0.70	0.125
RRAS	Related RAS viral (R-ras) oncogene homolog	3.67	3.46	0.67	0.591
CSN2	Beta-casein	7.23	7.08	0.48	0.729
SPP1	Osteopontin	5.06	5.09	−0.09	0.585
RAB10	Ras-related protein Rab-10	4.55	4.61	−0.20	0.720
EEF1A1	Elongation factor 1-alpha 1	4.17	4.23	−0.22	0.686
SAR1A	GTP-binding protein SAR1a	4.35	4.43	−0.26	0.737
LSS	Lanosterol synthase	4.27	4.39	−0.38	0.387
STX3	Syntaxin-3	4.64	4.76	−0.39	0.300
RAB5C	Ras-related protein Rab-5C	3.58	3.73	−0.51	0.850
CD9	CD9 antigen	5.79	5.99	−0.67	0.245
**XDH**	**Xanthine dehydrogenase/oxidase**	6.11	6.34	−0.76	0.047
ANXA2	Annexin A2	4.20	4.43	−0.76	0.081
**STOM**	**Erythrocyte band 7 integral membrane protein**	5.28	5.55	−0.88	0.027
ACTG1	**Actin, cytoplasmic 2**	4.71	4.98	−0.90	0.026
CD59	CD59 molecule, complement regulatory protein	6.12	6.40	−0.94	0.057
**FGFBP1**	**Fibroblast growth factor-binding protein 1**	4.60	4.90	−1.00	0.017
CIDEA	Cell death activator CIDE-A	4.57	4.87	−1.02	0.077
SAR1B	GTP-binding protein SAR1b	3.41	3.72	−1.03	0.280
HSP90AA1	Heat shock protein HSP 90-alpha	2.95	3.31	−1.19	0.510
**RAB1A**	**Ras-related protein Rab-1A**	4.68	5.06	−1.24	0.021
EHD4	EH domain-containing protein 4	3.53	3.91	−1.26	0.077
**BTN1A1**	**Butyrophilin subfamily 1 member A1**	6.80	7.32	−1.70	0.001
**PLIN2**	**Perilipin-2**	6.24	6.80	−1.87	0.001
**GNB1**	**Guanine nucleotide-binding protein G(I)/G(S)/G(T) subunit beta-1**	3.79	4.36	−1.87	0.006
**VAT1**	**Synaptic vesicle membrane protein VAT-1 homolog**	3.98	4.55	−1.88	0.003
**YWHAB**	14-3-3 protein beta/alpha	3.03	3.60	−1.91	0.432
**UBC**	**Polyubiquitin-C**	3.56	4.16	−2.02	0.001
**RAB18**	**Ras-related protein Rab-18**	5.29	5.93	−2.11	0.001
**MUC1**	**Mucin-1**	3.79	4.47	−2.26	0.046
**RAC1**	**Ras-related C3 botulinum toxin substrate 1**	3.85	4.60	−2.48	0.017
**RAB2A**	**Ras-related protein Rab-2A**	3.93	4.70	−2.57	0.000
**PIGR**	**Polymeric immunoglobulin receptor**	5.17	5.99	−2.73	0.000
**NUCB1**	**Nucleobindin-1**	2.58	3.54	−3.22	0.067
**YKT6**	**Synaptobrevin homolog YKT6**	3.68	4.64	−3.22	0.000
**FABP3**	**Fatty acid-binding protein, heart**	5.09	6.09	−3.33	0.001
**CSN1S1**	**Alpha-S1-casein**	6.69	7.79	−3.66	0.000
**ABCG2**	**ATP-binding cassette, sub-family G, member 2**	4.92	6.05	−3.75	0.000
**ACSL1**	**Acyl-CoA synthetase long-chain family member 1**	3.64	4.81	−3.89	0.000
**HSPA8**	**Heat shock cognate 71 kDa protein**	3.19	4.41	−4.06	0.000
**DHRS1**	**Dehydrogenase/reductase (SDR family) member 1**	3.77	5.03	−4.17	0.000
**CD36**	**Platelet glycoprotein 4**	4.78	6.25	−4.90	0.000
**GNB2**	**Guanine nucleotide-binding protein G(I)/G(S)/G(T) subunit beta-2**	3.40	5.34	−6.43	0.000
**IGL@**	**IGL@ protein**	4.39	6.49	−6.96	0.000
**MFGE8**	**Lactadherin**	4.46	6.72	−7.53	0.000

Tables 1 and 2 show the quantitative differences of common MFGM and milk serum proteins between human and bovine milk. Lipid synthesis and transport proteins, including fatty acid-binding protein, heart (FABP3), perilipin-2 (PLIN2), butyrophilin subfamily 1 member A1 (BTN1A1), lactadherin (MFGE8), and platelet glycoprotein 4 (CD36), were present at approximately 10–100 times higher abundance in bovine MFGM (*p* < 0.05). Serum albumin (ALB), monocyte differentiation antigen CD14 (CD14), alpha-lactalbumin (LALBA), lactoferrin (LTF), toll-like receptor 2 (TLR2), alpha-1-antitrypsin (SERPINA1), alpha-1-antichymotrypsin (SERPINA3), clusterin (CLU), and polymeric immunoglobulin receptor (PIGR) showed higher concentrations in human milk, especially for ALB, LTF, SERPINA3, and CD14, which were around 20–100 times higher in human milk serum (*p* < 0.05).

**Table 2:** Quantitative comparison of overlap human milk serum and bovine milk serum (Bold proteins are significantly different proteins by one-way ANOVA; the values are log10 average iBAQ values of proteins; Human milk samples were collected from 10 healthy mothers that were between 3 and 10 months in lactation; Bovine milk samples were collected from 30 clinically healthy cows that were between 3 weeks and 10 months in lactation; data was normally distributed)

Gene name	Protein name	HS	CS	Log2 (HS/CS)	*P* value
**ALB**	**Serum albumin**	7.30	5.27	6.75	0.000
**FASN**	**Fatty acid synthase**	4.38	2.40	6.59	0.000
**XDH**	**Xanthine dehydrogenase**	5.36	3.59	5.86	0.000
**SERPINA3**	**Alpha-1-antichymotrypsin**	5.44	4.02	4.72	0.000
**LTF**	**Lactoferrin**	7.17	5.84	4.44	0.002
**PRSS1**	**Alpha-trypsin chain 1**	6.74	5.42	4.39	0.000
**CD14**	**Monocyte differentiation antigen CD14**	5.52	4.24	4.23	0.000
**CLU**	**Clusterin**	5.74	4.64	3.66	0.002
**CSN2**	**Beta-casein**	7.80	6.79	3.34	0.002
LDHB	L-lactate dehydrogenase B chain	4.25	3.30	3.16	0.124
**SERPINA1**	**Alpha-1-antitrypsin**	5.38	4.62	2.52	0.007
**PIGR**	**Polymeric immunoglobulin receptor**	6.47	5.84	2.08	0.003
GC	Vitamin D-binding protein	5.20	4.64	1.89	0.026
LPL	Lipoprotein lipase	4.05	3.56	1.65	0.043
SPP1	osteopontin	6.35	5.95	1.34	0.107
HSPA8	Heat shock 70 kDa protein 8	3.89	3.51	1.25	0.277
LALBA	Alpha-lactalbumin	7.79	7.49	1.00	0.073
SERPINF2	Alpha-2-plasmin inhibitor	3.89	3.60	0.95	0.302
FABP3	Fatty acid-binding protein 3	5.33	5.13	0.66	0.359
B2M	Beta-2-microglobulin	5.47	5.36	0.37	0.779
BTN1A1	Butyrophilin subfamily 1 member A1	5.60	5.53	0.23	0.679
NUCB2	Nucleobindin 2	4.44	4.40	0.12	0.543
PLIN2	Perilipin-2	4.49	4.46	0.10	0.943
CSN1S1	Alpha-S1-casein	7.33	7.31	0.10	0.836
APOE	Apolipoprotein E	3.81	3.99	−0.59	0.285
SERPINC1	Antithrombin-III	3.86	4.05	−0.64	0.677
RAB18	Ras-related protein Rab-18	3.70	3.96	−0.86	0.725
MFGE8	Lactadherin	4.88	5.16	−0.94	0.402
NPC2	Epididymal secretory protein E1	4.49	4.89	−1.32	0.513
C3	Complement C3	4.56	4.96	−1.33	0.074
AZGP1	Zinc-alpha-2-glycoprotein	4.88	5.32	−1.47	0.197
AHSG	Alpha-2-HS-glycoprotein	4.32	4.83	−1.69	0.149
**IGL@**	**IGL@ protein**	6.19	6.76	−1.88	0.025
CFB	Complement factor B	2.66	3.27	−2.03	0.138
ORM1	Alpha-1-acid glycoprotein 1	4.94	5.56	−2.04	0.298
**FGFBP1**	**Fibroblast growth factor-binding protein 1**	3.69	4.64	−3.16	0.002
**LPO**	**Leucine-rich alpha-2-glycoprotein**	4.25	5.23	−3.26	0.004
**NUCB1**	**Nucleobindin 1**	4.05	5.35	−4.30	0.008
**IDH1**	**Isocitrate dehydrogenase 1**	3.00	4.48	−4.92	0.007

**Fig. 3:**
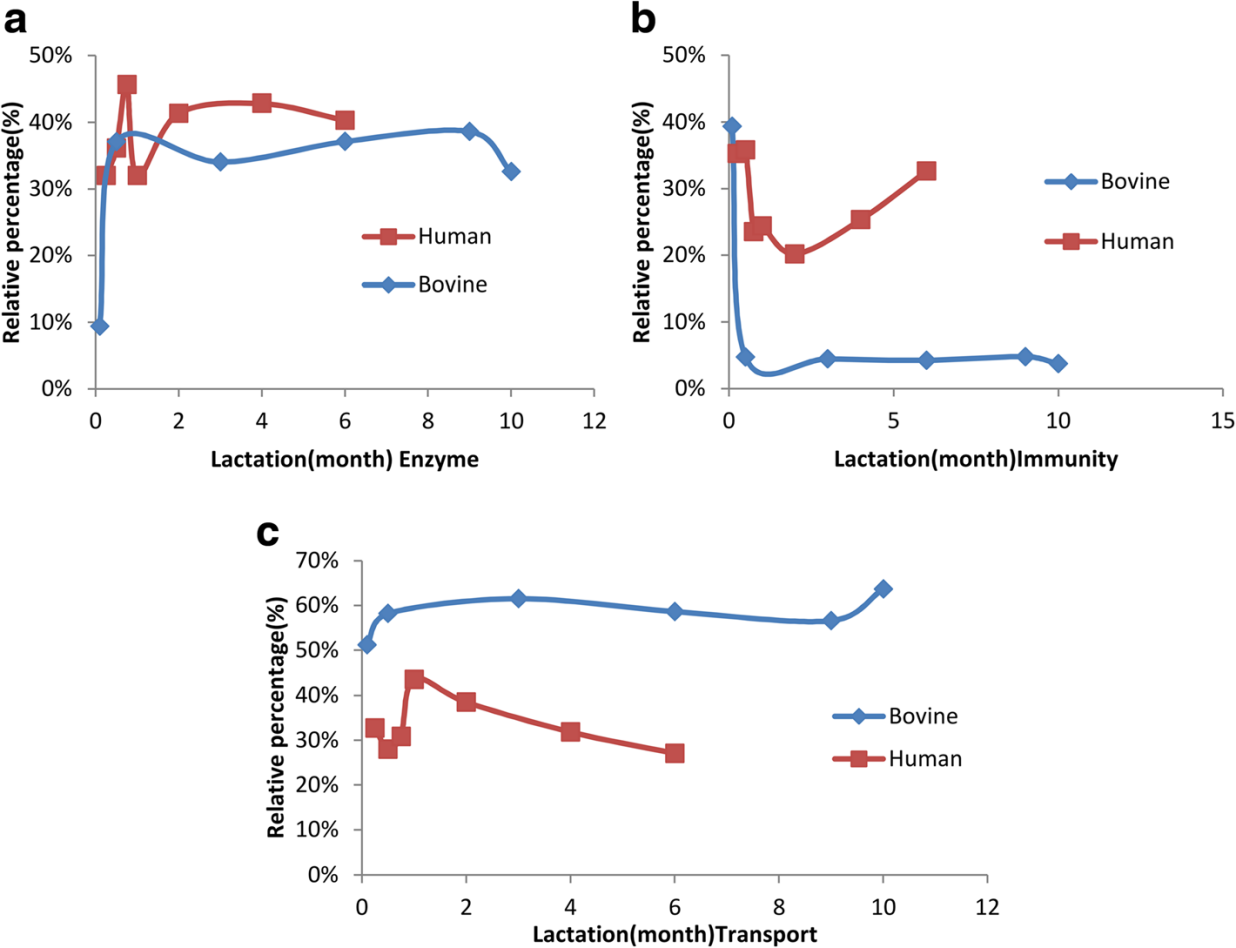
The relative changes of enzyme (**a**), immunity (**b**) and transport proteins (**c**) over lactation between human and bovine milk. The percentage is calculated through the total iBAQ value of proteins in each biological function group divided by the total iBAQ value of proteins belonging to these three groups

Since milk serum protein content is far higher than MFGM protein content [20], the quantitative changes over lactation were only determined for milk serum. A total of 299 proteins were quantified in bovine milk serum [13, 17] and 247 in human milk serum [16, 18] by FASP and dimethyl labelling combined with LC-MS/MS. There were 71 common proteins quantified in human and bovine milk serum, with 34 of them quantified in every time point over lactation. In addition to the high number of transport proteins in bovine milk serum, the concentration of the transport proteins (calculated based on the summed intensity based absolute quantification (iBAQ values)) was higher in bovine milk serum than human milk serum, whereas enzymes were higher in human milk serum (Figs. 2 and 3).

**Fig. 4:**
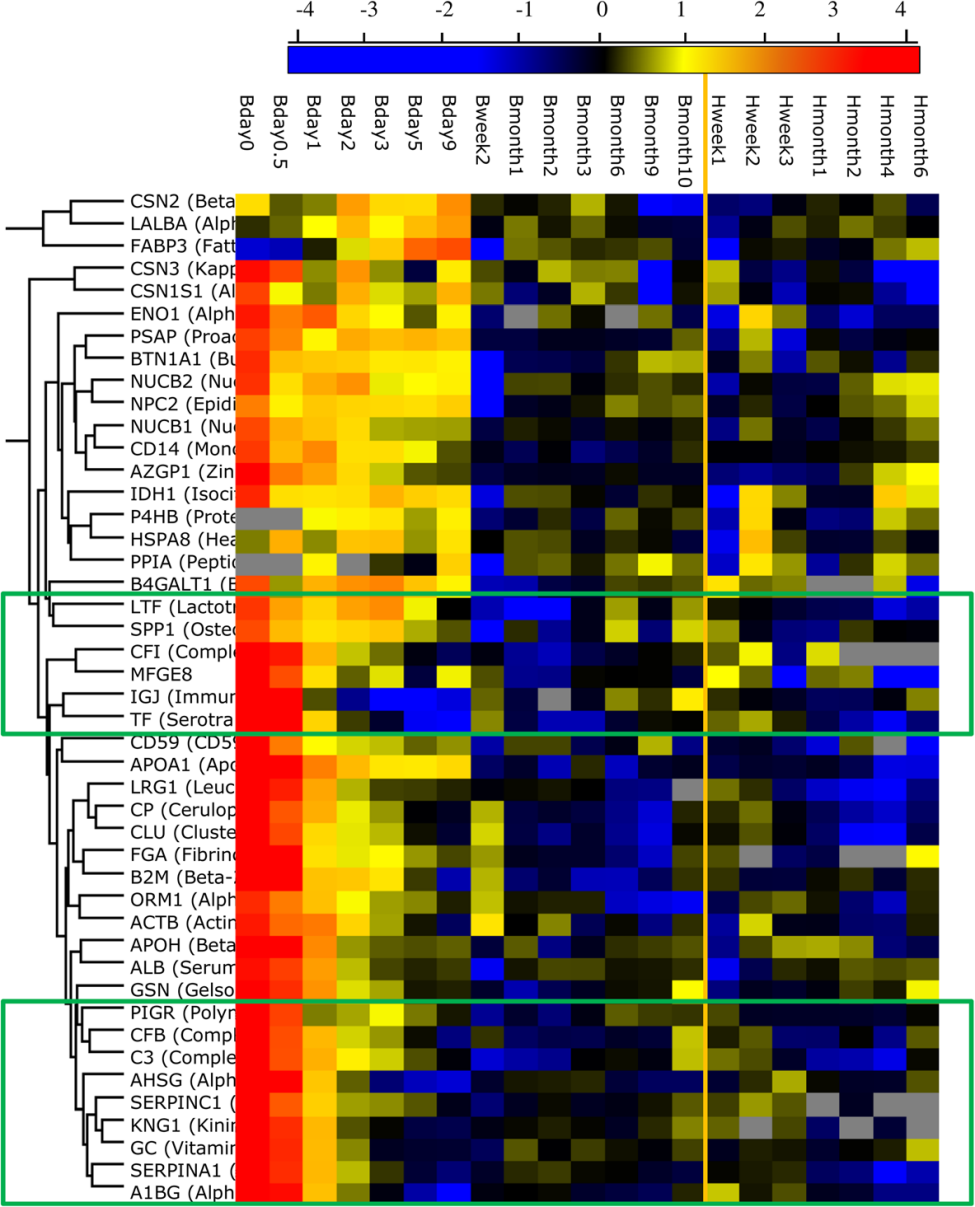
The changes in the protein concentration from human and bovine milk over lactation (B-bovine milk; H-human milk)

Although the biological function distribution were similar in the identified proteins between human and bovine (Fig. 2), the quantitative changes of these protein groups differed over lactation (Fig. 3). Immune-related protein group decreased during the course of lactation, whereas transport protein and enzymes increased (Fig. 3). Moreover, the changing rate of the protein with the same functionality differed between species (Fig. 3); for instance, immune-related proteins, LTF, complement C3 (C3), PIGR, and osteopontin (SPP1) decreased much faster in bovine milk serum compared to human milk serum (Fig. 4). The changes in immune-related proteins over lactation are important for two reasons. Firstly, immune-related proteins had relatively higher concentration in human milk than bovine milk. Secondly, these proteins play important roles in the protection of the neonate, which may therefore be proteins of interest for application in infant formula. Hierarchical clustering (Fig. 4) shows that these immune-related proteins are correlated to each other. In addition to the correlation of proteins related to complement and coagulation cascades, such as C3, complement factor I (CFI), complement factor B (CFB), SERPINA1, antithrombin-III (SERPINC1), and alpha-2-HS-glycoprotein (AHSG) discussed before [13], CLU, alpha-1-acid glycoprotein 1 (ORM1), actin, cytoplasmic 1 (ACTB), LTF, SPP1, and PIGR also showed close interactions in both human and bovine milk serum (Fig. 4).

In order to compare the common human and bovine milk serum proteome at the network level, we converted our expression data to co-expression networks, and obtained available protein-protein interaction data for both species. Analysis of protein-protein interaction data indicated that the milk serum proteins quantified in our study are highly connected. For example, 310 interactions were observed for 66 human milk serum proteins, which is roughly 50 times higher than the number of interactions expected for randomly chosen proteins. The observed high interaction density was statistically significant according to the statistical test provided by STRING (*p* < 10^−6^).

**Fig. 5:**
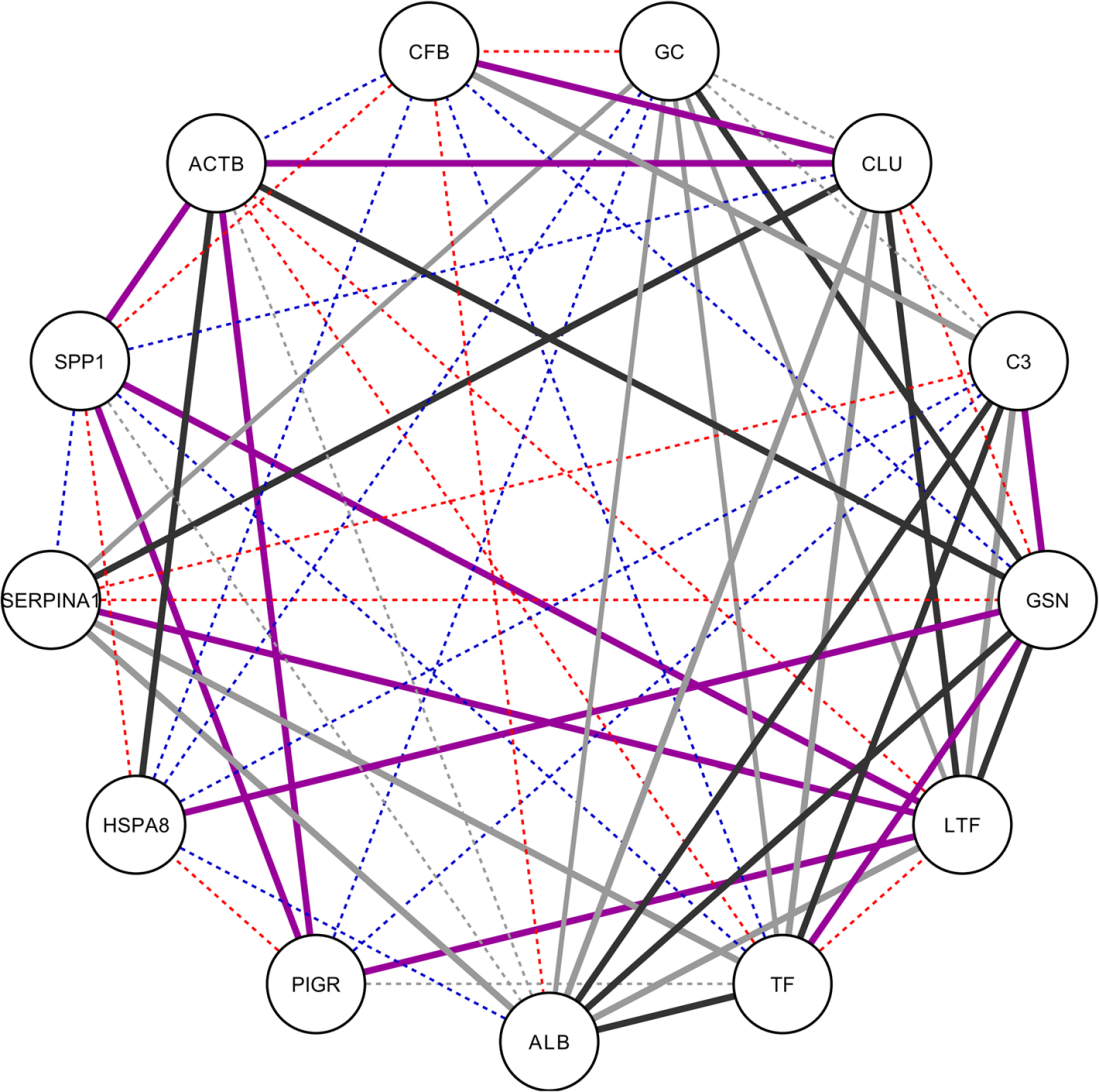
Common conserved core observed in network alignment between protein interaction network and bovine and human co-expression networks. Edge colors indicate in which of the networks interaction occur: only in human co-expression network (*red*), only in bovine co-expression network (*blue*), in both human and bovine co-expression network (*purple*), in human co-expression network and in protein interaction network (*black*), or other combinations of networks (*grey*). In addition, line type differentiates interactions occurring only in one network (*dashed lines*) from interactions occurring in multiple networks (*straight lines*)

Comparing the co-expression networks to each other, for 34 proteins quantified in every time point in both human and bovine milk serum, 18 were aligned to the equivalent protein in the other species. For these proteins, if they have expression similarity with another protein in human milk, it is likely that they also have expression similarity with that protein in bovine milk, and vice versa. For the other 16 proteins, network alignment indicated that this was not the case. In other words, these proteins have expression similarities with different proteins in human milk than in bovine milk, and are indicative of changes in the expression network between the two species (Fig. S1). The similarity between the human and bovine expression networks was also quantified using the correlation between the expression correlation coefficients. This resulted in a Pearson correlation coefficient of R = 0.23 (*p* < 10^−7^) between the expression Pearson correlation coefficients in human and bovine milk serum proteome. Comparing the human co-expression network with the protein interaction network, for 34 proteins, 17 were aligned to themselves. For these proteins, if they have expression similarity with another protein, it is likely that they also have protein interaction with that protein. Out of these, 13 proteins were among the above-mentioned 18 proteins which were aligned to the equivalent protein in the human-bovine co-expression network alignment. This indicates a common core of 13 proteins with relatively highly conserved interaction in each of the networks (Fig. 5). These include the immune-related C3, CLU, ACTB, SERPINA1, SPP1, PIGR, and LTF.

**Fig. 6:**
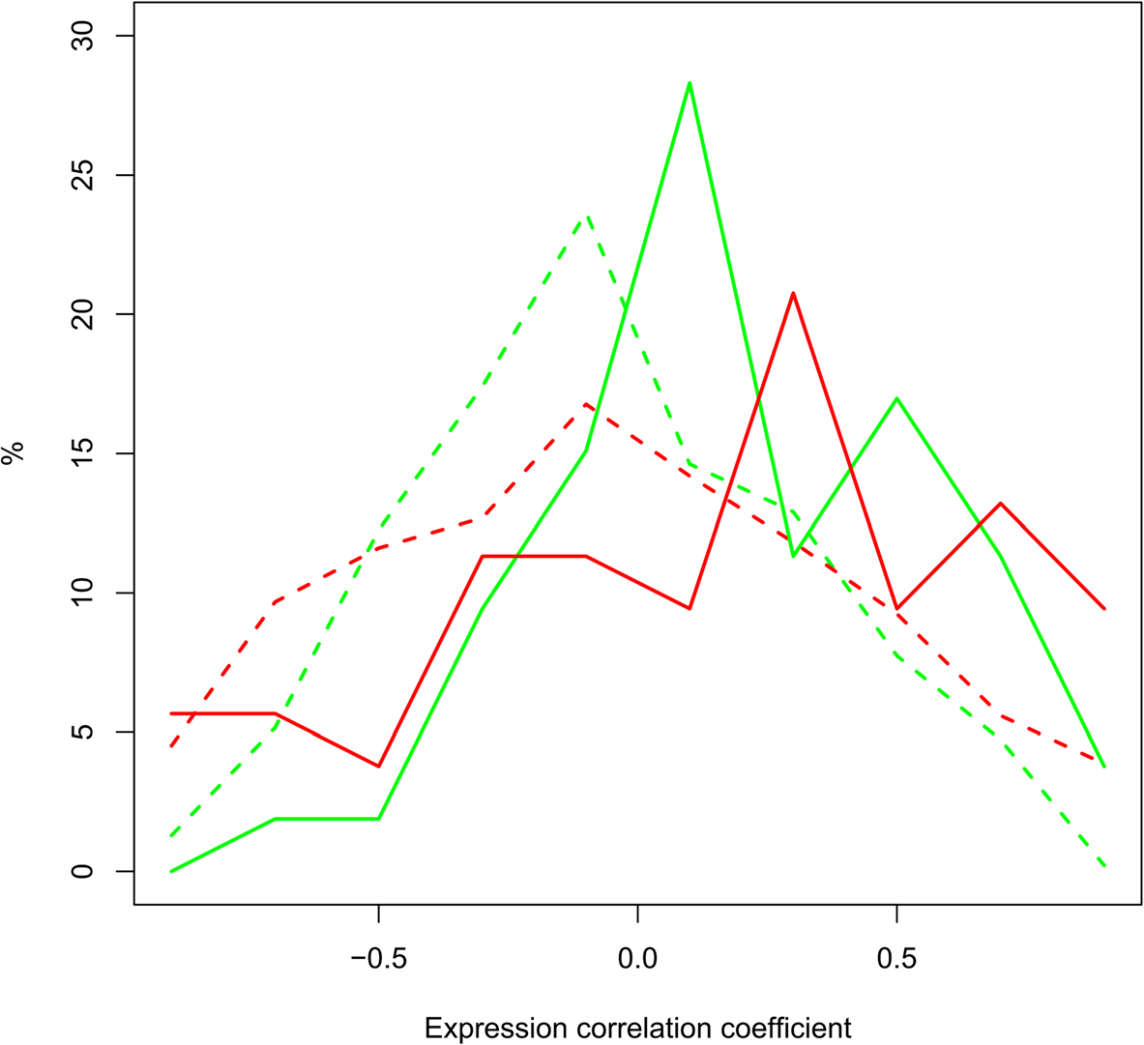
Proteins interacting in the protein-protein interaction network have higher expression correlation than proteins not interacting. Histogram of expression Pearson correlation coefficients for human (*green*) and bovine (*red*) protein pairs, separately for interacting (*straight lines*) and for non-interacting (*dashed lines*) protein pairs

The large agreement between co-expression networks and protein interaction networks observed based on the network alignment (Additional file 2: Figure S1 and Additional file 3: Table S2) was confirmed by analysing the relation between interaction status in the protein-protein interaction network, and expression correlation (both in human and bovine milk, Additional file 4: Table S3). The average expression correlation coefficient of non-interacting proteins is −0.06 +/−0.37, whereas for interacting proteins it is 0.18+/−0.37 (human) and 0.14+/−0.51 (bovine) respectively (Fig. 6). According to a Kolmogorov-Smirnov test, the differences between the distribution of correlation coefficients for interacting and for non-interacting proteins is significant: p ~ 10^−5^ (human interacting vs non-interacting) and p ~ 10^−3^ (bovine interacting vs non-interacting), respectively. Similarly, a Mann–Whitney *U* Test indicated that the means are significantly different (p ~ 10^−5^ for human interacting vs non-interacting and p ~ 0.005 for bovine interacting vs non-interacting).

## Discussion

Previous studies described some comparisons of the milk proteome between species [20–22]; however, they only used single samples, either mature milk collected at certain lactation stages or a pooled samples from different lactation stage. Also some reviews [23, 24] on milk proteome were based on single species, with no comparisons between different species. This is because the data they used are from different studies. Differences in lactation stage, differences in sample preparation methods, and differences in instruments make it difficult to compare the proteome between species at the same time points over lactation. This study was the first one to compare the changes of milk protein profile between human and bovine species at the same time points from colostrum to 6 months lactation by using the same sample preparation method and the same instrument. Our comparative analysis between the human and bovine lactation proteome was performed by reanalysing data from several of our previous studies [8, 13, 16–18]. The time-based comparison between human and bovine milk proteins, may help us to know better the differences in the needs between infants and calves. This may also provide guidance on the improvement of infant formula composition on different stages. Although the data interpretation of the lactation stage studies is limited by the small sample size (*n* = 4) for both species, the separate results for bovine and human milk are similar to previously published studies on the biological functions of bovine and human milk protein, with many proteins in both species contributing to nutrient transport and immune protection [23, 24]. The annotation in this study gives a first insight in the comparison in the milk proteomes between human and bovine and their changes over lactation. The network analysis indicates that both the biological functions and the concentration of proteins have similarities between human and bovine milk. The reanalysed results in the current study should contribute to better understanding of the differences and similarities in the biological functions and micronutrients between human and bovine milk proteome.

A total of 390 proteins were quantified using Maxquant in both human and bovine milk (Fig. 1), which is higher compared to our previous study [8]. However, the number of identified proteins were lower than that reported in previous studies [10, 20, 21, 23, 24]. First, this comparison is based on one study not on a large number of reviewed studies [23, 24]. Second, the lower number of identified proteins can be related to both the identification criteria (reducing identification confidence) and the extensive protein fractionation (increasing the proteome coverage but decreasing the precision of protein quantification), as discussed in our previous paper [16]. Moreover, Maxquant was time cost-efficient in protein quantification. This indicates the advantages of Maxquant in quantifying milk proteins. The higher number of quantified proteins in data set 1 than data set 2 can be related to the differences in the preparation methods. Label free was used for dataset 1 and dimethyl labelling was used for dataset 2. The shift from label free to dimethyl labelling in two studies is because dimethyl labelling is much more sensitive and precise to pick up small differences between two samples [25]. The lower number of quantified proteins in our studies compared with previous studies (e.g. 573 proteins from bovine milk [23], 1606 [22] and 976 [15] proteins from human milk) can be related to the extensive protein fractionation in these previous studies and less strict identification criteria as discussed in our previous paper [16].

The higher number of quantified MFGM proteins than milk serum proteins in both human and bovine (data set 1) is consistent with the numbers of identified proteins reported previously [8]. It is not surprising, as MFGM represent the epithelial cell, the place where the milk fat is synthesised and secreted [26, 27]. The low amount of transport proteins in human milk can be mainly related to the absence of the major transport protein β-lactoglobulin (LGB) in human milk [28], which is the most abundant protein in bovine milk serum. In addition, the lower concentration of lipid synthesis and secretion proteins in human milk (Table 1 and 2) also contributes to the relatively low amount of transport proteins in human milk.

The relative high amount of enzymes (Fig. 3) and the high biological enrichment (Additional file 1: Table S1) in human milk can probably be attributed to the immature gastrointestinal tract of infants at birth. Although the development of the gastrointestinal tract starts from the fetal stage, the maturation of the gastrointestinal digestive function is not complete at birth [29]. It experiences a dramatic switch in the nutrients from amniotic fluid before birth to colostrum after birth and the energy supply switches from glucose-dominated to lipid-dominated [30]. This transition requires the digestion of lipids and proteins prior to their absorption in the gastrointestinal tract [30]. The high abundant enzymes related to lipid and protein degradation in human milk, such as bile salt-activated lipase, lipoprotein lipase, trypsin, and cathepsin D [31], suggests that human milk itself contributes to the digestive capacity, thereby being able to more effectively deal with immature luminal digestion [32]. The differences in the dominant digestive enzymes between human milk (bile salt-activated lipase) and bovine milk (ribonuclease pancreatic), which have been discussed in our previous papers [17] may thus reflect the differences in the needs for support of the digestion system between infants and calves.

Previous studies have reported that calves develop their own immune system in a few weeks [33], whereas infants produce their own immunoglobulins only after 2 or 3 months [15]. The relatively higher amount and slower decrease of immune-related proteins in human milk (Fig. 3) may be related to the slower maturation of immune system in infants than calves, as hypothesized before [8]. This hypothesis is consistent with the in-depth comparison between human and bovine milk proteome (Tables 1 and 2, Figs. 3 and 4).

However, the common proteins present in human and bovine milk (Fig. 1) suggest the similarity in the milk proteome between human and bovine. Several common immune-related proteins in the network analysis of both biological functions and co-expression levels (Fig. 5) indicate the comparable immunological functions of milk proteins in protecting the neonate. In addition to the importance of dominant immune-related proteins, such as LTF and immunoglobulins discussed previously [14, 15], the low abundant immune-related proteins, including C3, CFB, SERPINA1, ACTB, and SPP1 (Fig. 5), play important roles in the immune system, especially innate immune system [10, 15]. The high abundance of innate immune-related proteins in early lactation (Fig. 4) may be due to its rapid reaction against broad groups of pathogens in the gastrointestinal tract of the neonate [8, 34], especially just after birth. SERPINA1 plays a dual role in regulating the complement and coagulation pathway [35], but also protecting the immune-related proteins against degradation during digestion. ACTB not only plays a role in the cell cytoskeleton but is also involved in innate immune response, according to research using a mice model [36]. SPP1 could protect the intestinal tract of infants against pathogens or bacteria, due to its cytokine-like properties and it being a key factor in the initiation of T helper 1 immune responses [37]. PIGR is the receptor of immunoglobulins A and M, facilitating their secretion in the mammary gland. The high correlation between SERPINA1, LTF, C3, ACTB, SPP1, and PIGR (Fig. 5) in both human and bovine milk reflects the interactions between innate and adaptive immune system and the complex nature of biological interrelationships between milk proteins in protecting the neonate.

The other common proteins in Fig. 5, LTF, TF, ALB, vitamin D-binding (GC), play roles in transport and delivery of nutrients through binding minerals, vitamins, fatty acid, steroids, glucocorticoid/progestin, and heme derivatives, and thus facilitate their uptake in the intestinal tract [38]. The correlation of these proteins in both human and bovine milk (Fig. 5) could be related to need for providing this range of micronutrients that are necessary for the growth of the neonate.

The distribution of expression correlation coefficients (Fig. 6) over lactation in both human and bovine milk proteome for protein pairs not interacting in the protein interaction network is shifted towards negative values compared to the distribution for protein pairs that are interacting. This suggests an interplay between protein-protein interactions and expression similarity. Such similarity between these different types of networks was also observed based on network alignment. In all mammals, milk provision is a complex process with changes in milk composition and interactions between parent and young beyond the straightforward nutritional function [39]. The similarity in the milk proteome may be related to their main functions in providing nutrients and protection to the neonate. The differences in the milk proteome between species may be due to their unique lactation strategies to accommodate reproductive success and adapt to the specific environment. This suggests an interplay between protein-protein interactions and expression similarity.

The comparison of the milk proteome between human and bovine over lactation provides more information on the similarity and differences of milk protein profile over lactation. This study can be used as a start point for further biological function investigation of proteins discussed in the paper. Proteins differing between human and bovine are interesting from an infant nutrition point-of-view. Further evaluation of the biological significance of these proteins, and on the feasibility of the application of such proteins in infant formula can be conducted. With respect to the proteins with high similarity based on the network alignment, they may still differ in digestibility or have different nutritional values due to the differences in amino acid sequence and post-translation modifications between species. Further studying this will contribute to a better understanding of protein functionality in human and bovine milk, and may provide guidance on the improvement of infant formula.

## Conclusions

The qualitative and quantitative differences between human and bovine milk proteome as well as the differences in the concentration changes over lactation help us to better understand the role of milk proteins in the development of the digestive and immune system of the neonates in general, including differences between infants and calves. The similarities in both protein-protein interaction network and expression correlation between human and bovine milk proteome indicates the importance of milk proteins in providing nutrients and protection to the neonate. This in-depth comparison between human and bovine milk contributes to a better understanding on the biological functions, especially immunological functions, of milk proteins between human and bovine.

## Methods

### Materials

In this study, we reanalysed the data collected on a single instrument [8, 13, 16–18] from both human and bovine milk proteome for an in-depth comparison throughout lactation.

#### Data set 1-Qualitative and quantitative differences between human and bovine milk proteome study

This data is based on the study of Hettinga, et al. [8]. Human milk was collected from 10 healthy mothers between 3 and 10 months in lactation. Samples of 10 mL were collected and frozen for later analysis. After thawing, the 10 samples were pooled. One bovine tank milk sample was collected from the university farm “De Ossekampen” in Wageningen, The Netherlands, which was milk from 30 clinically healthy cows which were between 3 weeks and 10 months in lactation.

#### Data set 2-The comparison in the changes of human and bovine milk proteome over lactation

This data set is based on our previous studies [13, 16–18]. Human milk samples were collected from women who gave birth at the obstetric department in VU medical center (VUmc) in Amsterdam. All women who delivered singleton term infants (gestational age 37–42 weeks) were eligible for this study. Women with haemolysis elevated liver enzymes, low platelet syndrome, history of breast surgery, and (gestational) diabetes mellitus were excluded. The samples collected at week 1, 2, 3, 4, 8, 16, 24 were used for this study. Approximately 5–10 mL was collected in a polypropylene bottle after 1 min of pumping for every sample. and stored at −18 °C immediately afterwards.

Bovine milk was collected from four healthy cows in a farm in Zaffelaere, Belgium. The cows were milked using an automatic milking system. Samples were collected from day 0 to the end of lactation. Samples collected at day 0, 0.5, 1, 2, 3, 5, 9, 14, month 1, 2, 3, 6, 9 and the latest time point of the lactation (10 months for cow 1, 11 months for cow 2 and 12 months for cow 3, the latest time point was missed for cow 4) were used for this study. The samples were frozen immediately at −20 °C after collection and transferred frozen to the laboratory for further analysis.

### Methods

#### Milk serum separation

The separation of milk serum was performed according to a previous study [8]. The samples were centrifuged at 1,500 × g for 10 min at 10 °C (Beckman coulter Avanti J-26 XP centrifuge, rotor JA-25.15). The milk fat was removed and the obtained supernatant was transferred to the ultracentrifuge tubes followed by ultracentrifugation at 100,000 × g for 90 min at 4 °C (Beckman L-60, rotor 70 Ti). After ultracentrifugation, samples were separated into three phases. The top layer was remaining milk fat, the middle layer was milk serum (with some free soluble caseins), and the bottom layer (pellet) was casein. Milk serum was used for filter aided sample preparation as described below after the measurement of protein content by the BCA protein assay (Fisher Scientific).

#### Proteomic techniques

##### Filter aided sample preparation

Filter aided sample preparation (FASP) was performed as previously described [40]. Milk serum samples (20 μL), including samples of each time point and pooled samples of each included woman, were diluted in 100 mM Tris/HCl pH 8.0 + 4% SDS + 0.1 M Dithiotreitol (SDT-lysis buffer) to get a 1 μg/μL protein solution. Samples were then incubated for 10 min at 95 °C, and centrifuged at 18407 g for 10 min, after cooling down to room temperature. Twenty μL of each sample were directly added to the middle of 180 μL 0.05 M iodoacetamide/100 mM Tris/HCl pH 8.0 + 8 M urea (UT) in a low binding Eppendorf tube and incubated for 10 min while mildly shaking at room temperature. The sample was transferred to a Pall 3 K omega filter (10–20 kDa cutoff, OD003C34; Pall, Washington, NY, USA) and centrifuged at 15871 g for 30 min. Three repeated centrifugations at 15871 g for 30 min were carried out after adding three times 100 μL UT. After that, 110 μL 0.05 M NH_4_HCO_3_ in water (ABC) were added to the filter unit and the samples were centrifuged again at 15871 g for 30 min. Then, the filter was transferred to a new low-binding Eppendorf tube. One hundred μL ABC containing 0.5 μg trypsin were added followed by overnight incubation at room temperature. Finally, the sample was centrifuged at 15871 g for 30 min, and 3.5 μL 10% trifluoroacetic acid (TFA) were added to the filtrate to adjust the pH value of the sample to around 2. These samples were ready for dimethyl labeling.

##### Dimethyl labeling

The dimethyl labeling was carried out by on-column dimethyl labeling according to [22]. The trypsin digested samples of pooled milk serum from each individual mothers and cows collected at the different time points were labeled with light reagent (the mix of CH_2_O and cyanoborohydride), whereas trypsin digested milk serum samples of the individual mothers and cows at each time point were labeled with heavy reagent (the mix of CD_2_O and cyanoborohydride). Stage tips containing 2 mg Lichroprep C18 (25 um particles) column material (C18+ Stage tip) were made in-house. The C18+ Stage tip column was washed 2 times with 200 μL methanol. The column was conditioned with 100 μL of 1 mL/L formic acid in water (HCOOH) after which samples were loaded on the C18+ Stage tip column. The column was washed with 100 μL 1 mL/L HCOOH, and then slowly flushed with 100 μL labeling reagent (0.2% CH_2_O or CD_2_O and 30 mM cyanoborohydride in 50 mM phosphate buffer pH 7.5) in about 10 min. The column was washed again with 200 μL 1 mL/L HCOOH. Finally, the labeled peptides were eluted with 50 μL of 70% acetonitrile/30% 1 mL/L HCOOH from the C18+ Stage tip columns. The samples were then dried in a vacuum concentrator (Eppendorf Vacufuge®) at 45 °C for 20 to 30 min until the volume of each sample decreased to 15 μL or less. The pairs of light dimethyl label and heavy dimethyl label were then mixed up and the volume was adjusted to exactly 100 μL by adding 1 mL/L HCOOH. These samples were ready for analysis by LC-MS/MS.

##### LC-MS/MS

Eighteen μL of the trypsin digested and dimethyl labeled milk fractions were injected on a 0.10 × 30 mm Magic C18AQ 200A 5 μm beads (Michrom Bioresources Inc., USA) pre-concentration column (prepared in house) at a maximum pressure of 270 bar. Peptides were eluted from the pre-concentration column onto a 0.10 × 200 mm Prontosil 300-3-C18H Magic C18AQ 200A 3 μm analytical column with an acetonitrile gradient at a flow of 0.5 μL/min, using gradient elution from 8 to 33% acetonitrile in water with 0.5 v/v% acetic acid in 50 min. The column was washed using an increase in the percentage acetonitrile to 80% (with 20% water and 0.5 v/v% acetic acid in the acetonitrile and the water) in 3 min. A P777 Upchurch microcross was positioned between the pre-concentration and analytical column. An electrospray potential of 3.5 kV was applied directly to the eluent via a stainless steel needle fitted into the waste line of the microcross. Full scan positive mode FTMS spectra were measured between m/z 380 and 1400 on a LTQ-Orbitrap XL (Thermo electron, San Jose, CA, USA). CID fragmented MS/MS scans of the four most abundant doubly- and triply-charged peaks in the FTMS scan were recorded in data-dependent mode in the linear trap (MS/MS threshold = 5.000).

#### Data analysis

The acquired datasets were analyzed by using MaxQuant (Version 1.5.2.8, http://www.maxquant.org/) and the built-in Andromeda search engine with a UniProt human and bovine database (http://www.uniprot.org/; accessed March 2012). The search parameters were as follows: variable modifications of protein N-terminal acetylation and methionine oxidation, and fixed modification of cysteine carbamidomethylation. The minimum peptide length was set to 7 amino acids and a maximum of 2 missed cleavages was allowed for the search. Trypsin/P was selected as the semi-specific proteolytic enzyme. The global false discovery rate (FDR) cut off used for both peptides and proteins was 0.01 [41]. Label-free quantitation was performed in MaxQuant. To further improve the quantification accuracy, only the razor/unique peptides were used for quantitative calculations. The other parameters used were the default settings in MaxQuant software for processing MS/MS data.

All known contaminants (i.e. keratins, trypsin), and proteins detected in less than half of the samples, were removed from each sample set of proteins identified. The origin and function of the identified proteins was taken from UniProtKB (http://www.uniprot.org/; accessed March 2012) for recommended protein name, gene name, and protein function. It was verified that the human and bovine proteins with the same protein name were orthologous using a reciprocal best BLAST hit approach. DAVID Bioinformatics Resource 6.7 (https://david.ncifcrf.gov/) was used for protein biological function classification and protein group enrichment. Protein concentrations were calculated as the average of all peptide peak intensities from five replicates divided by the number of theoretically observable tryptic peptides (intensity based absolute quantification, or iBAQ, [42, 43]). Perseus software v.1.2.0.16 (Martinsreid, Germany) was used to test for hierarchical clustering and significant differences between species. Hierarchical clustering in Perseus software was used for clustering proteins identified in both human and bovine milk based on their relative abundance. This procedure is performing hierarchical clustering of rows (proteins) and columns (samples) and produces a visual heat map representation of the clustered matrix. The ratios between the concentration found in human milk (milk fat globulin membrane-MFGM and serum) and bovine milk (MFGM and serum) were calculated as the difference (on ^10^log scale) of the iBAQ value of the human MFGM versus the bovine MFGM and human serum versus bovine serum. ANOVA was applied to compare MFGM and serum in both species, and the p-values obtained were adjusted with false discovery rate (FDR)-based correction in order to account for the effect of multiple comparisons.

Protein-protein interactions for proteins in both human and bovine milk proteome were obtained from STRING [44]. In order to interpret the interaction density (number of observed interactions divided by total possible number of interactions) of milk proteins, this density was compared with the interaction density of all human/bovine STRING proteins. A statistical test for the significance of the observed high density in the milk proteome was performed using the approach provided by STRING [45].

For co-expression network analysis, a cutoff of 0.3 on the absolute value of the Pearson correlation was applied, in order to get a number of interactions in the co-expression networks that would be comparable to that in the STRING interaction networks. Pinalog [46] was used to align different networks to each other, taking into account both sequence similarity between proteins and topological similarity (i.e. similarity of interaction partners for each protein). For visualization, VANLO [47] and Cytoscape [48] were applied. Comparison of distributions with Kolmogorov-Smirnov test was performed using the R-function ks.test.

## Additional files

Additional file 1: Table S1. The biological functional enrichment of immunity, transport and enzyme protein groups in both human and bovine milk. (DOCX 13 kb)
Additional file 2: Figure S1. Network alignment between bovine (red) and human (green) co-expression networks. Equivalent nodes are connected by thin straight lines and are at comparable positions in the two networks. (TIF 18148 kb)
Additional file 3: Table S2. Alignment between bovine and human co-expression networks. (DOCX 14 kb)
Additional file 4: Table S3. Alignment between protein interaction network and human co-expression network. (DOCX 15 kb)

## Abbreviations

ACTB: Actin, cytoplasmic 1
AHSG: Alpha-2-HS-glycoprotein
ALB: Serum albumin
BTN1A1: Butyrophilin subfamily 1 member A1
CD14: Monocyte differentiation antigen CD14
CD36: Platelet glycoprotein 4
CEL: Bile salt-activated lipase
CFB: Complement factor B
CFI: Complement factor I
CLU: Clusterin
FABP3: Fatty acid-binding protein, heart
FASP: Filter-aided sample preparation
FDR: False discovery rate
GC: Vitamin D-binding
GO: Gene ontology
iBAQ Value: intensity based absolute quantification
LALBA: Alpha-lactalbumin
LGB: β-lactoglobulin
LPL: Lipoprotein lipase
LTF: Lactoferrin
MFGE8: Lactadherin
MFGM: Milk fat globule membrane
ORM1: Alpha-1-acid glycoprotein 1
PIGR: Polymeric immunoglobulin receptor
PLIN2: Perilipin-2
PRSS1: Alpha-trypsin chain 1
RNASE1: Pancreatic ribonuclease 1
RNASE4: Ribonuclease 4
SERPINA1: Alpha-1-antitrypsin
SERPINA3: Alpha-1-antichymotrypsin
SERPINC1: Antithrombin-III
SPP1: Osteopontin
TLR2: Toll-like receptor 2
