# Supplementary material for: An interactomics overview of the human and bovine milk proteome over lactation
Source: Proteome Sci. 2017 Jan 5;15:1. doi: 10.1186/s12953-016-0110-0 (PMC5267443; doi:10.1186/s12953-016-0110-0)
Supplement: Additional file 1: Table S1. — The biological functional enrichment of immunity, transport and enzyme protein groups in both human and bovine milk. (DOCX 13 kb) [file 12953_2016_110_MOESM1_ESM.docx]

| Biological function | Bovine_Enrichment Score | Bovine_Pvalue | Human_Enrichment Score | Human_Pvalue |
| --- | --- | --- | --- | --- |
| Immunity | 7.57873 | 7.60E-09 | 14.32 | 1.40E-15 |
| Transport | 2.56 | 5.00E-04 | 2.94 | 9.20E-06 |
| Enzyme | 0.87 | 7.60E-03 | 8.99 | 4.40E-12 |

Table S1. The biological functional enrichment of immunity, transport and enzyme protein groups in both human and bovine milk
