# Supplementary figures and images for: An interactomics overview of the human and bovine milk proteome over lactation
Source: Proteome Sci. 2017 Jan 5;15:1. doi: 10.1186/s12953-016-0110-0 (PMC5267443; doi:10.1186/s12953-016-0110-0)

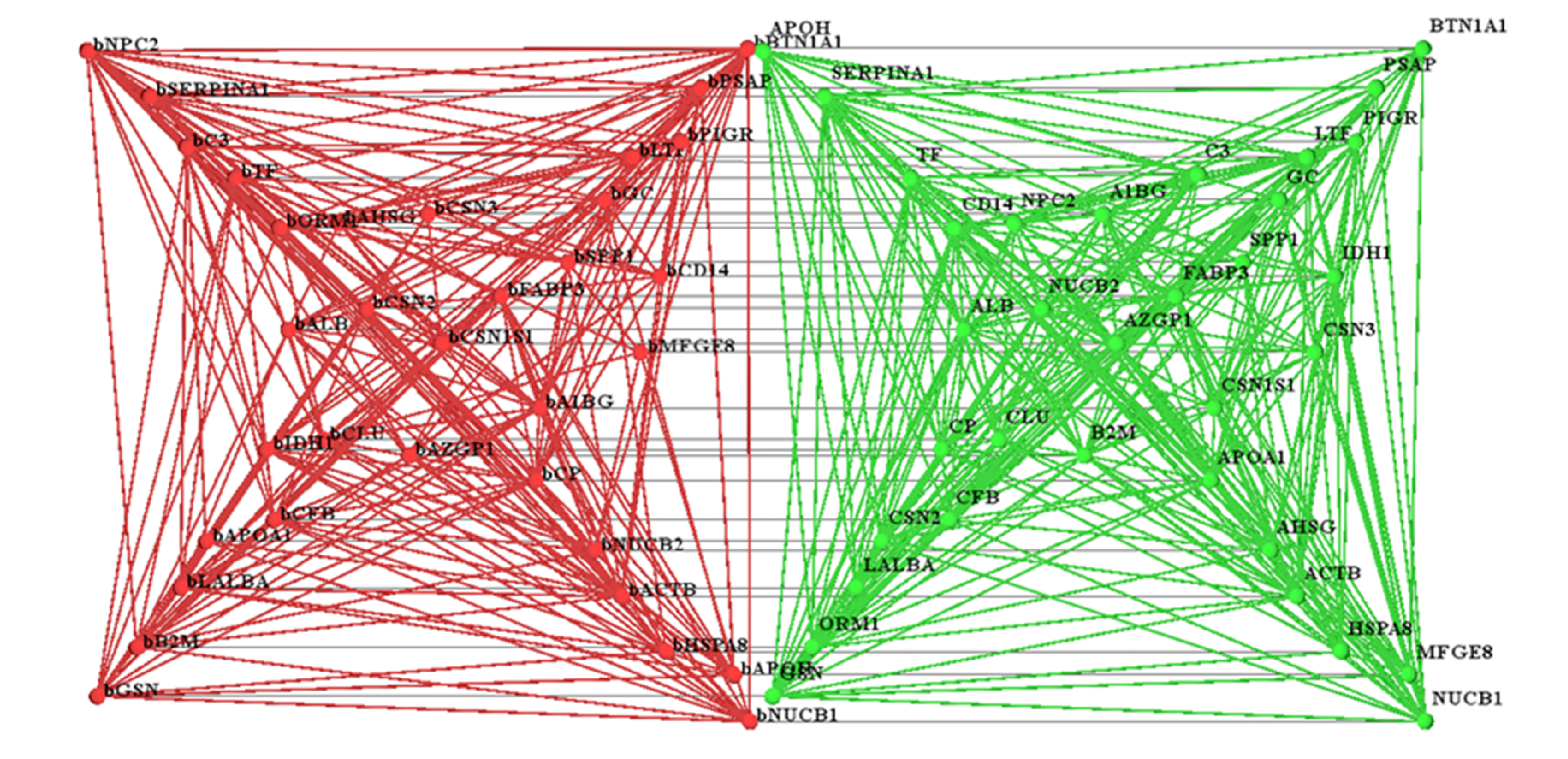

Supplement: Additional file 2: Figure S1. — Network alignment between bovine (red) and human (green) co-expression networks. Equivalent nodes are connected by thin straight lines and are at comparable positions in the two networks. (TIF 18148 kb) [file 12953_2016_110_MOESM2_ESM.tif]
