# Supplementary material for: An interactomics overview of the human and bovine milk proteome over lactation
Source: Proteome Sci. 2017 Jan 5;15:1. doi: 10.1186/s12953-016-0110-0 (PMC5267443; doi:10.1186/s12953-016-0110-0)
Supplement: Additional file 3: Table S2. — Alignment between bovine and human co-expression networks. (DOCX 14 kb) [file 12953_2016_110_MOESM3_ESM.docx]

Supporting Information Table 1. Alignment between bovine and human co-expression networks.

| Bovine protein | Human protein |
| --- | --- |
| TF | TF |
| GSN | GSN |
| ACTB | ACTB |
| ALB | ALB |
| BTN1A1 | BTN1A1 |
| CFB | CFB |
| GC | GC |
| HSPA8 | HSPA8 |
| LTF | LTF |
| PSAP | PSAP |
| PIGR | PIGR |
| C3 | C3 |
| A1BG | CSN1S1 |
| AHSG | NPC2 |
| APOH | MFGE8 |
| B2M | ORM1 |
| CLU | CLU |
| CP | APOA1 |
| CSN1S1 | AZGP1 |
| CSN2 | NUCB2 |
| FABP3 | FABP3 |
| IDH1 | CP |
| LALBA | LALBA |
| NPC2 | APOH |
| NUCB2 | AHSG |
| SERPINA1 | SERPINA1 |
| SPP1 | SPP1 |
| MFGE8 | CSN3 |
| ORM1 | CD14 |
| CSN3 | A1BG |
| NUCB1 | NUCB1 |
| APOA1 | CSN2 |
| AZGP1 | B2M |
| CD14 | IDH1 |
