# Supplementary material for: An interactomics overview of the human and bovine milk proteome over lactation
Source: Proteome Sci. 2017 Jan 5;15:1. doi: 10.1186/s12953-016-0110-0 (PMC5267443; doi:10.1186/s12953-016-0110-0)
Supplement: Additional file 4: Table S3. — Alignment between protein interaction network and human co-expression network. (DOCX 15 kb) [file 12953_2016_110_MOESM4_ESM.docx]

Supporting Information Table 2. Alignment between protein interaction network and human co-expression network.

| Protein interaction network | Co-expression network |
| --- | --- |
| ALB | ALB |
| TF | TF |
| SERPINA1 | SERPINA1 |
| CLU | CLU |
| CP | CP |
| GC | GC |
| LTF | LTF |
| ACTB | ACTB |
| HSPA8 | HSPA8 |
| C3 | C3 |
| CFB | CFB |
| GSN | GSN |
| IDH1 | IDH1 |
| A2M | AZGP1 |
| APOA1 | NPC2 |
| APOE | LALBA |
| APOH | APOH |
| KNG1 | B2M |
| B2M | NUCB1 |
| ENO1 | AHSG |
| FN1 | CD14 |
| AHSG | NUCB2 |
| FGA | MFGE8 |
| FGG | A1BG |
| SERPINC1 | PSAP |
| SPP1 | SPP1 |
| CSN1S1 | CSN1S1 |
| GGT1 | ORM1 |
| APOA4 | APOA1 |
| LALBA | CSN3 |
| CFI | CSN2 |
| PIGR | PIGR |
| ITIH4 | BTN1A1 |
| UBC | FABP3 |
